# Calcium-Binding Capacity of Centrin2 Is Required for Linear POC5 Assembly but Not for Nucleotide Excision Repair

**DOI:** 10.1371/journal.pone.0068487

**Published:** 2013-07-02

**Authors:** Tiago J. Dantas, Owen M. Daly, Pauline C. Conroy, Martin Tomas, Yifan Wang, Pierce Lalor, Peter Dockery, Elisa Ferrando-May, Ciaran G. Morrison

**Affiliations:** 1 Centre for Chromosome Biology, School of Natural Sciences, National University of Ireland Galway, Galway, Ireland; 2 Anatomy, School of Medicine, National University of Ireland Galway, Galway, Ireland; 3 Bioimaging Center, University of Konstanz, Konstanz, Germany; 4 Department of Physics, Center for Applied Photonics, University of Konstanz, Konstanz, Germany; University of Louisville, United States of America

## Abstract

Centrosomes, the principal microtubule-organising centres in animal cells, contain centrins, small, conserved calcium-binding proteins unique to eukaryotes. Centrin2 binds to xeroderma pigmentosum group C protein (XPC), stabilising it, and its presence slightly increases nucleotide excision repair (NER) activity in vitro. In previous work, we deleted all three centrin isoforms present in chicken DT40 cells and observed delayed repair of UV-induced DNA lesions, but no centrosome abnormalities. Here, we explore how centrin2 controls NER. In the centrin null cells, we expressed centrin2 mutants that cannot bind calcium or that lack sites for phosphorylation by regulatory kinases. Expression of any of these mutants restored the UV sensitivity of centrin null cells to normal as effectively as expression of wild-type centrin. However, calcium-binding-deficient and T118A mutants showed greatly compromised localisation to centrosomes. XPC recruitment to laser-induced UV-like lesions was only slightly slower in centrin-deficient cells than in controls, and levels of XPC and its partner HRAD23B were unaffected by centrin deficiency. Interestingly, we found that overexpression of the centrin interactor POC5 leads to the assembly of linear, centrin-dependent structures that recruit other centrosomal proteins such as PCM-1 and NEDD1. Together, these observations suggest that assembly of centrins into complex structures requires calcium binding capacity, but that such assembly is not required for centrin activity in NER.

## Introduction

As the principal microtubule organising centre in animal somatic cells, centrosomes play important roles in controlling cell shape and polarity, as well as directing the formation of the mitotic spindle through establishing its poles. Mitotic centrosomes have a distinctive ultrastructure, consisting of two centrioles, cylindrical structures composed of microtubules, within the pericentriolar material (PCM) (reviewed in 1–3). Centrosome composition alters as the organelles duplicate during the cell cycle, with the centrioles disengaging at the end of mitosis, serving as templates for new centriole formation during S phase, and ultimately moving apart to form the spindle at the onset of the next mitosis. Centrioles also bind to the plasma membrane and act as the basal bodies for cilia or flagella.

Centrins are small, evolutionarily conserved, calcium-binding proteins that are crucial for basal body assembly and/or function in lower eukaryotes [4–6]. They also localise to centrosomes and basal bodies in mammalian cells [7–12], although the bulk of cellular centrin is not centrosomal [12]. Centrins have a distinctive structure that derives from their containing two pairs of EF-hands, helix-loop-helix structures that bind calcium, separated by a linker region [13,14]. Although all centrins are related to the calcium-binding protein, calmodulin, two major subfamilies are recognised. Members of one subfamily are closer to the originally-cloned budding yeast CDC31p than are members of the other, which are more homologous to *Chlamydomonas* centrin [5,15,16]. Humans have 4 centrin genes: the ubiquitously-expressed centrin3 is of the CDC31p family [15] and centrin1, centrin2 and centrin4 of the *Chlamydomonas* centrin family. *CETN1* is restricted to certain cell types in its expression pattern and *CETN4* is a pseudogene in human cells, while *CETN2* is expressed ubiquitously in humans [17,18].

A number of studies have addressed vertebrate centrin functions in the centrosome using siRNA and gene targeting approaches (reviewed by [19]). While some reports describe markedly impaired centriole biogenesis in the absence of human centrin2 [20,21], this effect was not observed by other groups [22,23], although a minor delay in the assembly of CP110 into procentrioles was noted [24]. Our own experiments with gene targeting in chicken DT40 cells demonstrated intact centriole formation and centrosome functions in the absence of all 3 centrin isoforms [25]. Loss of centrin2 does, however, reduce primary ciliogenesis in human cells [21,26] and, in zebrafish embryos, leads to developmental abnormalities that phenocopy those seen in ciliopathies [27]. Together, these data support a crucial role for centrins in ciliogenesis, rather than in centriole assembly.

Another important activity of centrin2 lies in nucleotide excision repair (NER), a DNA repair process that removes bulky DNA adducts, such as the 6-4 photoproducts and cyclobutane pyrimidine dimers generated by ultraviolet (UV) light (reviewed by [28–30]). Centrin2 co-fractionates with xeroderma pigmentosum group C protein (XPC) and its interactor, HRAD23B, key proteins that direct the initial DNA damage recognition step of an NER pathway termed global genome repair [31,32]. The role played by centrin2 in NER is unclear: in vitro NER can be carried out in its absence, although it increases NER activity somewhat [32–35]. Nevertheless, centrin2 moves to the nucleus after UV irradiation in an XPC-dependent manner and is required for efficient repair of UV-induced DNA lesions in both human and chicken cells [25,36].

An important question is the regulation of the centriolar localisation of centrins and their roles in NER. Centrin2, in particular, has been shown to be multiply phosphorylated [4,12,37], so here we use site-directed mutagenesis of phospho-target sites to explore the relationship between centriole localisation, NER capacity and the ability of centrin2 to form linear structures. We have also mutated key residues of the centrin2 EF-hand domains to impair Ca^2+^ binding capacity. Mutants of interest were expressed in the centrin-null DT40 cell line that we have previously described [25], so that their cellular distribution would not be influenced by endogenous centrin isoforms. We found that centrin2 localisation to the centrosome is reduced in certain phospho-site mutants and abrogated by disruption of the EF-hands, although NER activity was still rescued in cells that expressed these mutants. These findings indicate that the initial steps of NER are not dependent on the phospho-regulation of centrin2, or its localisation at the centriole. We also found that EF-hand function is crucial for assembly of centrins into linear structures in cells that overexpressed POC5, which may suggest a role for calcium binding by centrin2 in the formation of complex centrin-containing structures.

## Results

**Figure 1:**
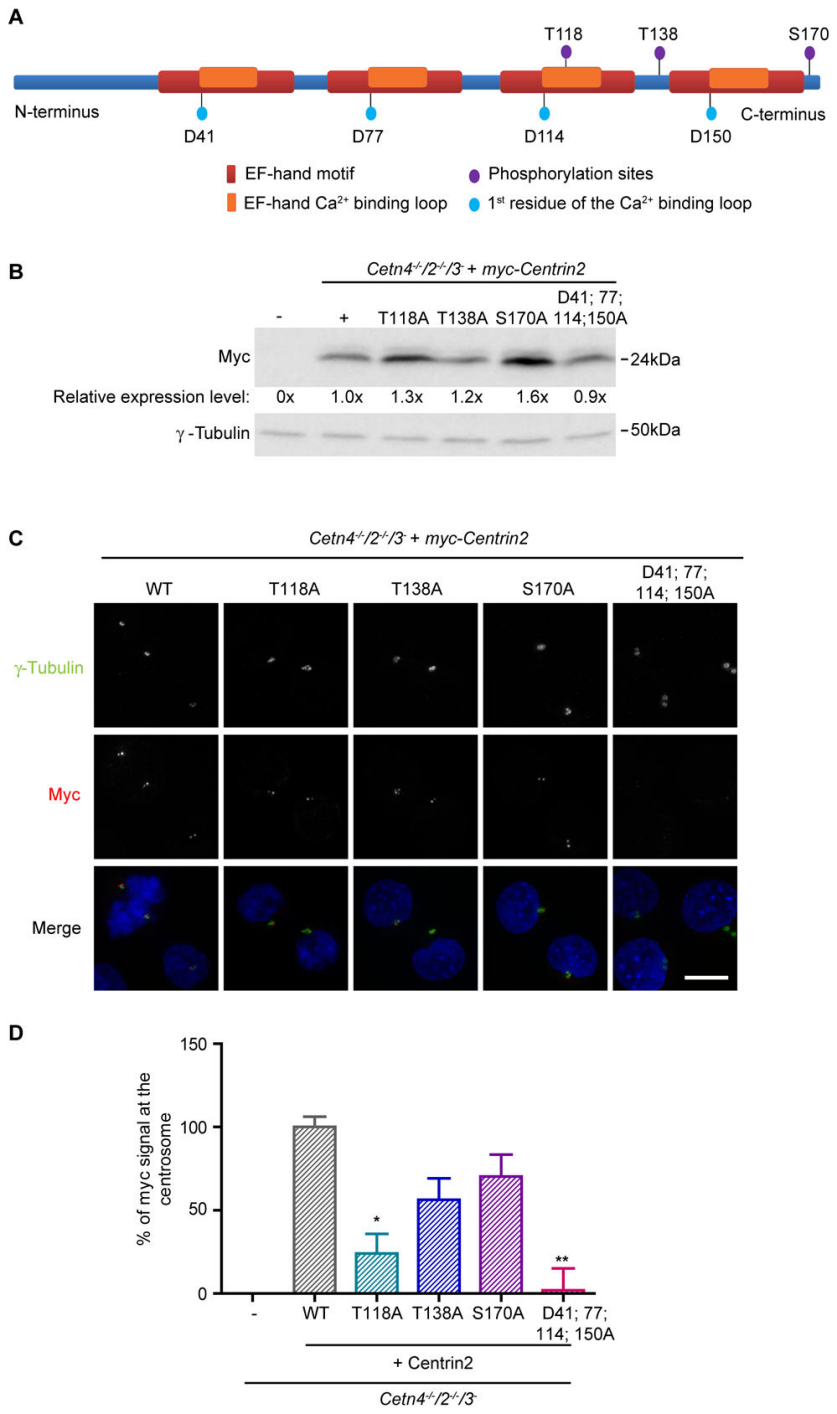
Localisation of centrin2 to the centrosome. **A**. Bar diagram of chicken centrin2, showing the relative positions of the phospho-sites and EF-hand residues that were mutated in this study. **B**. Immunoblot showing the relative expression levels of the myc-centrin2 transgenes in the clones used in this study. Numerical values show the mean of 3 separate experiments. **C**. Immunofluorescence micrograph showing the localisation of wild-type and the indicated mutants of centrin2 after stable transfection of *Cetn4* ^*-/-*^ */2* ^*-/-*^ */3* ^*-*^ DT40 cells. DNA was labelled with DAPI (blue). Scale bar, 5 µm. **D**. Quantitation of the relative centrin2 signal detected at the centrosome (co-localisation with γ-tubulin). Histogram shows the mean percentage + s.d. of the control signal seen in at least 1000 cells in 3 separate experiments. The control was *Cetn4* ^*-/-*^ */2* ^*-/-*^ */3* ^*-*^ DT40 cells that stably expressed wild-type myc-centrin2. *, *P* <0.05; **, *P* <0.01 compared to controls by Kruskal-Wallis test and Dunn’s multiple comparison test. Size markers are indicated at right.

We set out to identify the regulatory elements of centrin2 that dictate its localisation to the centrosome. Using the NetPhos 2.0 prediction software (http://www.cbs.dtu.dk/services/NetPhos/ [38]), we found 8 predicted phosphorylation sites that are conserved between human and chicken centrin2: S20; T26; T94; T118; S122; T138; S170; Y172. Of these, three have been reported as target sites for phosphorylation: T118 by MPS1, T138 by CK2 and S170 by Aurora-A [24,39,40]. We used site-directed mutagenesis to mutate each of these 3 residues to alanine (A) in a myc-centrin2 construct which expresses a protein that reproduces the subcellular localisation of endogenous centrin2 and rescues the NER defect of centrin-deficient cells [25]. We also converted to alanine the first amino acid of the loop region of each EF-hand, which is typically an aspartate residue and which has been shown to be crucial for Ca^2+^ binding [41–43]. These mutations were combined into a construct that would express a centrin unable to bind calcium, myc-centrin2-D41A; D77A; D114A; D150A (‘D41; 77; 114; 150A’). A diagram of centrin2 and the residues mutated in this study is shown in Figure 1A.

**Figure 2:**
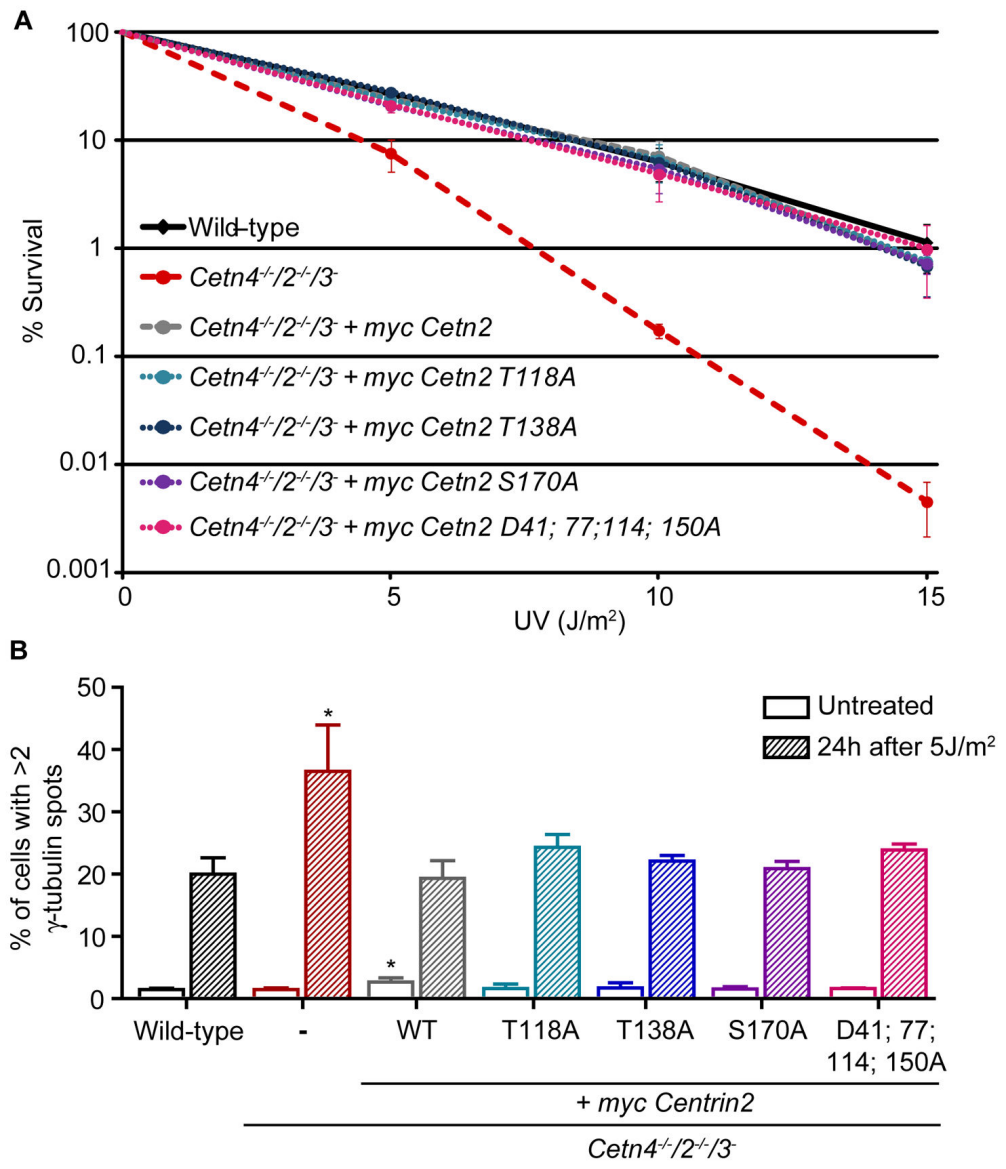
NER functions of centrin2 are independent of centrosome localisation. **A**. Clonogenic survival of cells of the indicated genotype after exposure to the indicated doses of UV irradiation. Datapoints represent the mean survival + s.d. of 3 separate experiments. **B**. Centrosome amplification in cells of the indicated genotype, quantitated before or 24h after 5J/m^2^ UV irradiation by microscopy for γ‐tubulin. Histogram shows the mean + s.d. of cells with amplified centrosomes from 3 separate experiments in which at least 2000 cells were quantitated. *, *P* <0.05 compared to wild-type cells by Kruskal-Wallis test and Dunn’s multiple comparison test.

We performed transient transfections of these constructs in centrin-deficient DT40 cells. We observed no impact on centrosome numbers in transfected cells and, while centrosomal recruitment of the T138A and S170A forms of centrin2 was comparable to wild-type, we observed a greatly-reduced centrosomal localisation of the T118A and D41; 77; 114; 150A mutants (data not shown). To quantitate this localisation more robustly, we generated cell lines that stably expressed myc-centrin2 on the centrin-deficient background, analysing clones whose transgene expression level was as close as possible to that of the wild-type control (Figure 1B). As shown in Figure 1C, D, the T138A and S170A mutant forms localised robustly to the centrosome, suggesting that centrin2 targeting to the centrosome is independent of phosphorylation at these residues. However, the centrosomal localisation of the T118A mutant centrin2 was greatly reduced and the calcium-binding mutant failed almost entirely to localise to the centrosome. Using these mutants, we next assessed whether a centrosomal localisation determines the activity of centrin2 in NER. In clonogenic survival assays, we found that all the centrin2 phospho-site mutants and the calcium-binding mutant were as efficient as wild-type centrin2 in rescuing the hypersensitivity to UV irradiation seen in centrin-deficient cells (Figure 2A). We also found that a hyperamplification of centrosome number seen in centrin-deficient cells after UV treatment [25] was rescued by expression of any the mutants under investigation to the same extent as by wild-type centrin2 (Figure 2B). These results strongly suggest that calcium binding and centrin phosphorylation at the residues we examined are dispensable in NER and that centrin2 activity in NER is independent of its centrosomal localisation.

**Figure 3:**
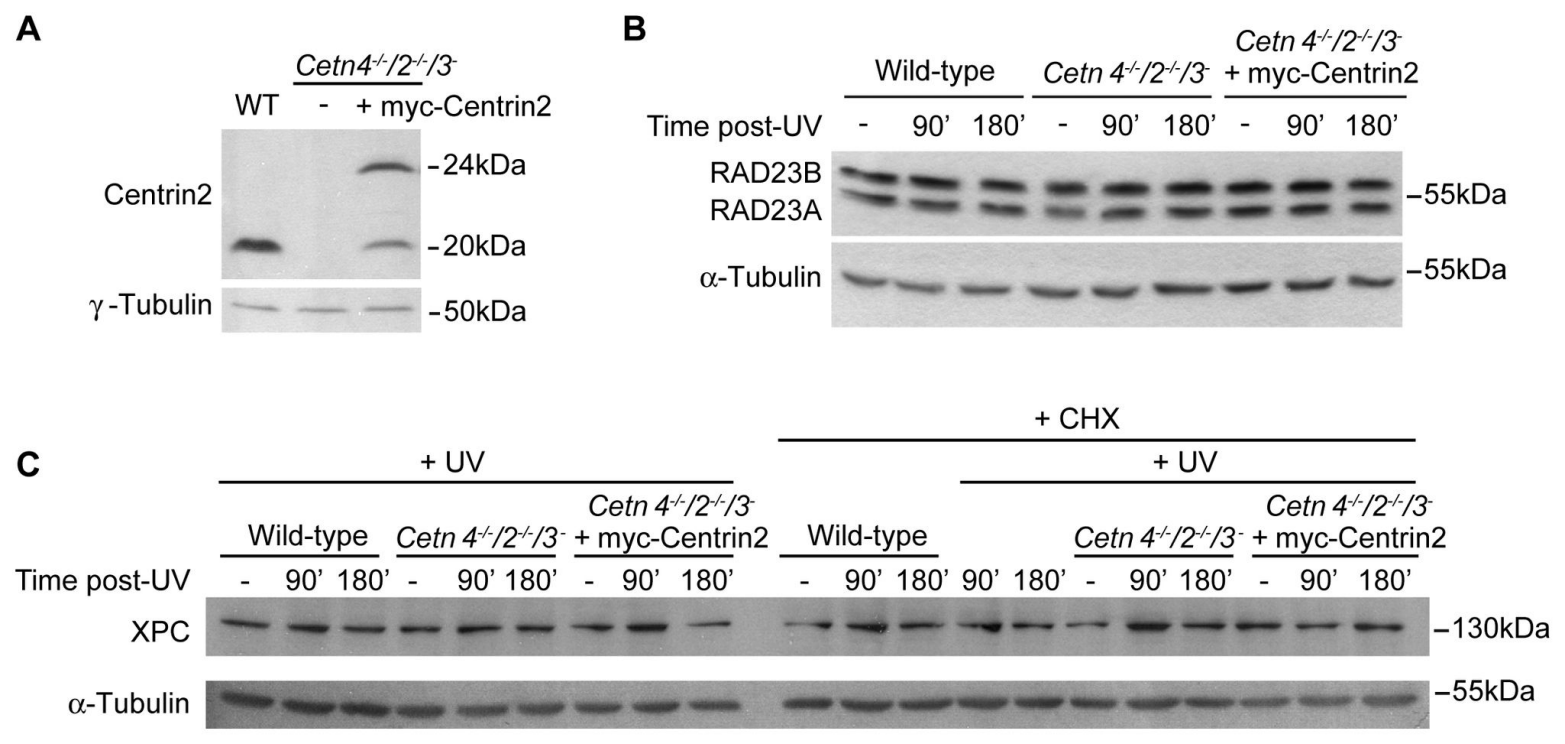
Centrin deficiency does not impact on protein levels of the XPC-RAD23 complex. **A**. Immunoblot shows the expression level of centrin2 in cells of the indicated genotype. Two bands are seen in the myc-centrin2 rescue clone due to a second methionine in the centrin2 transgene coding sequence serving as a second potential start site. Immunoblots show the levels of **B**. RAD23A and RAD23B; **C**. XPC before and at the indicated times after 20J/m^2^ UV irradiation in wild-type, centrin-deficient and rescued DT40 cells. The rescue clone was that used in Figure 1B. Where indicated, cycloheximide (CHX) was added at 100µg/ml at time 0. Results shown are representative of 3 experiments. α- and γ-Tubulin were used as loading controls. Size markers are indicated at right.

It has been proposed that centrin2 increases the stability of the XPC-HRAD23B complex [32,44]. Therefore, we tested whether XPC or RAD23 levels were affected by the absence of centrin2. Centrin2 expression levels were determined in wild-type DT40 cells, the null cell line and in the wild-type rescue clone used in Figure 1A (Figure 3A). We then used commercial antibodies raised against human XPC and RAD23B to analyse the levels of their chicken homologues by immunoblotting before and after a high dose of UV irradiation. As shown in Figure 3B and 3C, the levels of chicken XPC and RAD23 did not vary substantially in the absence of centrins, either before or after irradiation. A requirement for XPC degradation in efficient NER has been described [45]. As any XPC degradation following UV irradiation might be masked by new protein synthesis, we treated cells with cycloheximide to block translation. However, we did not observe any UV-induced changes in XPC levels (Figure 3A). Given the rapid proliferation of DT40 cells, it is possible that there may be a short timecourse for XPC degradation and restoration in NER that precluded our seeing such changes, even in the presence of cycloheximide. In any case, we did not find that centrin deficiency potentiated a major alteration in cellular levels of XPC or RAD23. Next, we investigated the centrin dependency of XPC localisation. We cloned the coding sequence of the chicken *XPC* orthologue and transiently expressed GFP-cXPC in wild-type and centrin-deficient cells. In both wild-type and *Cetn*-deficient cells, GFP-cXPC localised to the interphase cell nucleus but was dispersed throughout the cytoplasm in mitotic cells (data not shown). These observations suggest that the absence of centrins does not affect the normal nuclear localisation of cXPC.

**Figure 4:**
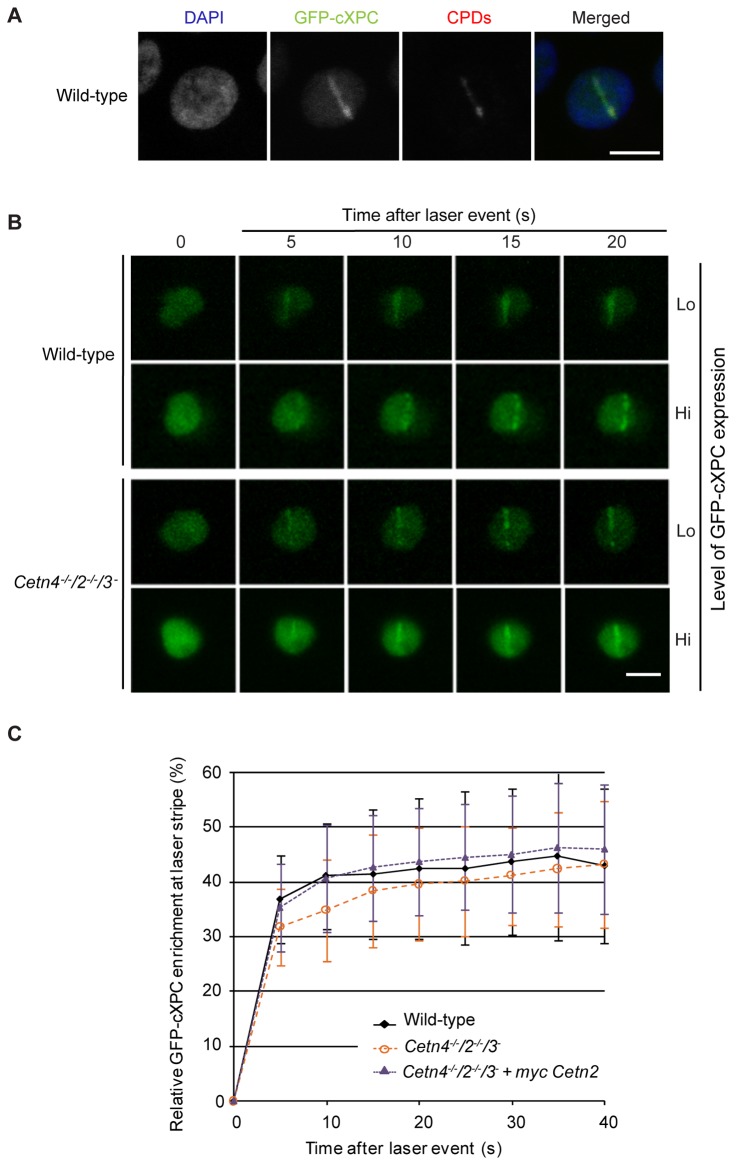
Recruitment of XPC to DNA damage occurs in the absence of centrin. **A**. Immunofluorescence micrograph shows the recruitment of GFP-cXPC to laser-induced DNA damage. Antibodies to cyclopyrimidine dimers were used to visualise the damaged region (red), which colocalises with GFP-cXPC (green). DNA was labelled with DAPI (blue). Scale bar, 5 µm. **B**. Live-cell visualisation of GFP-cXPC recruitment to DNA damage in cells of the indicated genotype. Micrographs show the GFP channel at different times after irradiation. Different GFP-cXPC expression levels were examined for recruitment kinetics. Scale bar, 5 µm. **C**. Quantitation of GFP-cXPC recruitment to the stripe of laser-induced DNA damage. Curve shows the mean increase over background + s.d. for measurements taken in wild-type (*N*=36), centrin-deficient (*N* =34) and rescued (*N* =31) cells.

To test if centrin2 is required for the recruitment of XPC to UV-induced DNA lesions, we used a pulsed multi-photon laser at 775nm to induce sub-nuclear DNA lesions in wild-type and centrin-deficient cells that expressed GFP-cXPC. This methodology allowed us to monitor the time-dependent mobilisation of GFP-cXPC by live-cell imaging at laser-induced photo-lesions (CPDs and 6-4 photoproducts) within spatially-defined laser stripes [46,47]. We first validated this procedure in DT40 cells by fixing laser-irradiated wild-type cells and staining them with an antibody against CPDs. Mobilisation of GFP-cXPC to the irradiated region can be seen as a stripe of co-localisation with the CPD signal within the nucleus (Figure 4A). Next, we monitored the recruitment/enrichment kinetics of GFP-cXPC at laser-induced lesions in both wild-type and centrin-deficient cells, with high or low levels of GFP-cXPC expression (Figure 4B). We then determined the kinetics of GFP-cXPC enrichment in wild-type, centrin-deficient and centrin2 rescue cell lines. Previous studies using this procedure in human fibroblasts have shown an almost immediate relocalisation of XPC-GFP following DNA damage [46]. As shown in Figure 4C, laser irradiation of both wild-type and centrin-deficient cells resulted in a rapid accumulation of GFP-cXPC at the laser stripes. This accumulation stabilised 15-40 seconds after the laser event, plateauing more rapidly in wild-type and centrin2 rescue cells than in centrin-deficient cells. However, taking the standard deviation into consideration, the minor kinetic difference observed here cannot explain the severe UV sensitivity seen in the absence of centrins in a long-term colony forming assay and the significantly slower DNA repair kinetics of UV-induced photoproducts [25]. We conclude that the nuclear localisation, DNA damage recognition and binding abilities of cXPC are not affected by centrin deficiency, suggesting that the critical role for centrin2 in NER lies downstream of the XPC-mediated recognition step.

Reverse genetic experiments in human and zebrafish have provided strong evidence for a role for centrin2 in ciliogenesis [21,26,27]. Unfortunately, since DT40 cells are a B-cell lymphoma line and therefore do not assemble primary cilia [48], we have been unable to explore this activity of centrin2 directly. We instead explored whether centrin2 deficiency or inability to localise to centrosomes might affect proteins at the distal end of centrioles. We found that IFT88 (Polaris), a component of the intraflagellar transport machinery that also localises to the distal end [49] was robustly recruited to centrin-deficient centrosomes, showing a non-significant increase to 106 + 9% of wild-type levels of centrosomal IFT88 fluorescence intensity (N = 3; > 1000 cells/ experiment; data not shown).

**Figure 5:**
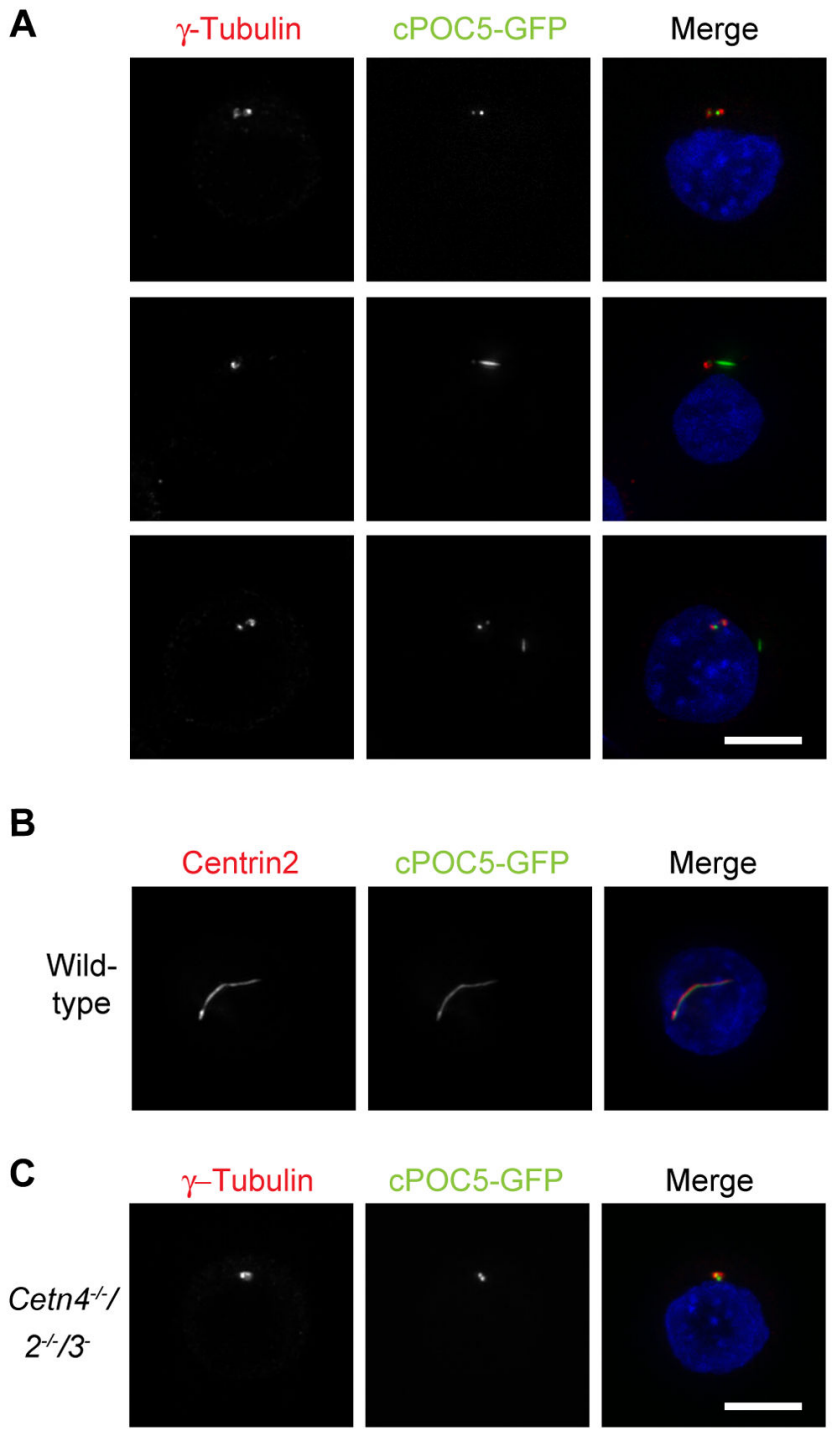
cPOC5 overexpression induces linear structures. **A**. Immunofluorescence micrograph shows examples of the structures induced by transient transfection of DT40 cells with a cPOC5-GFP expression construct. GFP is shown in the green channel, centrosomes were identified by γ-tubulin staining (red) and DNA was visualised with DAPI (blue). Micrographs are representative of results obtained in at least 2 separate experiments. Scale bar, 5 µm. **B**. Centrin colocalisation with cPOC5-GFP structures was observed by microscopy for centrin2 (red), with cells otherwise being transfected, fixed and stained as for **A**. **C**. Centrosomal localisation of cPOC5-GFP was observed in centrin-deficient cells. Cells were transfected, fixed and stained as for **A**.

A second protein that we examined was POC5, a centrosomal protein which has been shown to directly interact with centrins in human cells and which localises to the distal lumen of centrioles, where it is required for centriole elongation [50]. We cloned and transiently expressed a GFP fusion of the chicken POC5 orthologue of hPOC5. 97% of wild-type cells with cPOC5-GFP expression showed a clear centriolar or centrosomal localisation (*N*=3 experiments in which at least 150 cells were scored). Strikingly, approximately 40% of GFP-positive cells also contained defined, linear structures that at a first glance resembled primary cilia, even though in 26% of cells with these structures, the assemblies did not always localise at the centrosome (Figure 5A, B). Endogenous centrin2 consistently showed complete co-localisation with the cPOC5-GFP signal, regardless of whether a linear structure was present in the cell (Figure 5C). Interestingly, in 94% of the centrin-deficient transfectants, cPOC5-GFP localised exclusively at the centrioles or centrosomes without assembling the defined, linear structures observed in wild-type cells.

**Figure 6:**
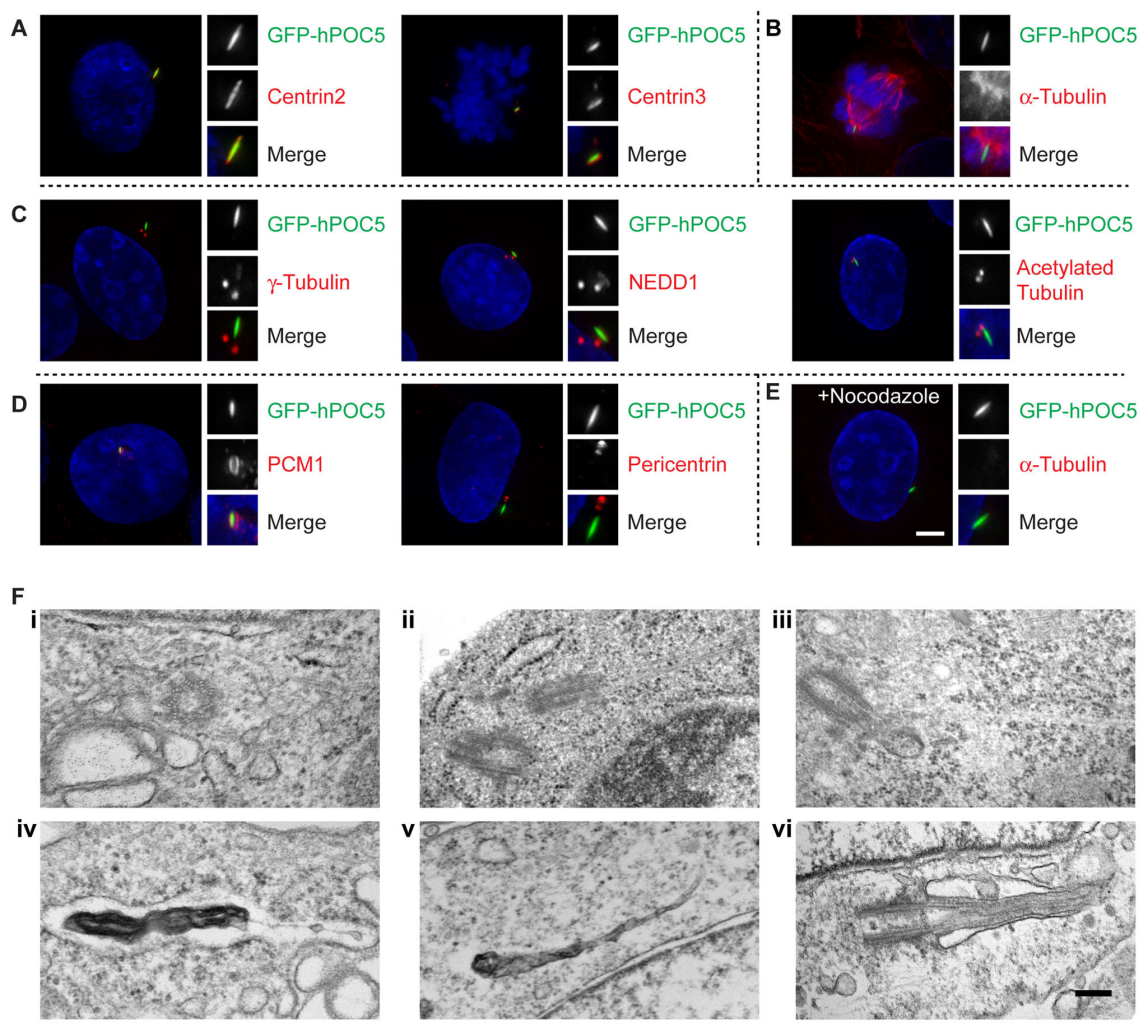
Characterisation of linear, hPOC5-induced structures. Immunofluorescence micrographs show examples of the structures induced by stable expression of hPOC5-GFP in U2OS cells. GFP is shown in the green channel, with relevant markers in red and DNA visualised with DAPI (blue). Blow-ups (2.5x) show the hPOC5-induced structures. Micrographs are representative of results obtained in at least 3 separate experiments. Scale bar, 5 µm. A. Centrin2 and centrin3 (red) localise at the hPOC5-induced structures. B. hPOC5-induced structures are observed in a proportion of mitotic cells but do not contain α-tubulin. C. hPOC5-induced structures contain γ-tubulin and NEDD1, but not acetylated tubulin (stabilized microtubules). D. PCM1, but not pericentrin, is seen at hPOC5-induced structures. E. hPOC5 structures are resistant to microtubule depolymerisation. Cells were treated with 2µg/ml nocodazole for 2h before fixation. F. Electron micrographs of (**i, ii, iv, v**) U2OS cells with stable expression of hPOC5 showing (**i**) normal cross-sectional centriole structure; examples of elongated centriolar microtubules in (ii) U2OS and (iii) DT40 cells that overexpressed POC5; (**iv**)**, (v)** examples of electron-dense, linear aggregates (**vi**). Micrograph shows a primary cilium in hTERT-RPE1 cells. Scale bar, 200 nm.

We also tested if POC5 overexpression induced the formation of similar structures in human cells. We cloned both hPOC5 isoforms 1 and 2 [50] and expressed them in hTERT-RPE1 cells. Approximately 35% of cells that overexpressed hPOC5 isoform 2 showed a defined, linear structure that co-localised with centrins (Figure 6A), as did 7% of cells that overexpressed isoform 1. We speculated that this phenomenon might be analogous to the reported centriole over-elongation observed upon POC1, CPAP (CenpJ) overexpression and/or CP110 depletion [51–55]. However, further characterisation of these structures in U2OS cells showed that they do not contain α-tubulin or stabilised microtubules, as determined by the analysis of glutamylated or acetylated tubulin, or the cilia/ centrosome components IFT88, Ninein, rootletin, centrobin and ODF2 (Figure 6B, C Figure S1). Nevertheless, members of the γ-tubulin ring complex (γ-TuRC), such as γ-tubulin and NEDD1, localised to approximately 50% of the POC5-induced structures (*N* > 30 cells; Figure 6C Figure S1). In addition, we also found PCM-1, but not pericentrin, surrounding a similar percentage of the structures (*N* > 30 cells; Figure 6D). Furthermore, we found that these structures remained intact, even after cells had been treated with nocodazole, suggesting no requirement for microtubules in the maintenance of these structures (Figure 6E).

We next examined the centrosomes in hPOC5-expressing cells by transmission electron microscopy (TEM). We found 2 examples of long microtubular structures arising from the centrosomes/centrioles, but the remaining centrosomes that we analysed were of the normal dimensions and showed the typical nine sets of triplet microtubules (*N* > 60 cells in both DT40 and U2OS; Figure 6F(i-iii)). However, in a substantial number of cells, we found what appeared to be protein aggregates composing somewhat linear structures, presumably not connected with centrosomes and quite distinct from primary cilia structures (Figure 6F(iv-v)). A micrograph of a primary cilium from hTERT-RPE1 cells is shown in Figure 6F(vi) for comparison. We conclude that POC5 overexpression induces the formation of a linear, non-centriolar structure that can recruit centrins and some components of the PCM.

**Figure 7:**
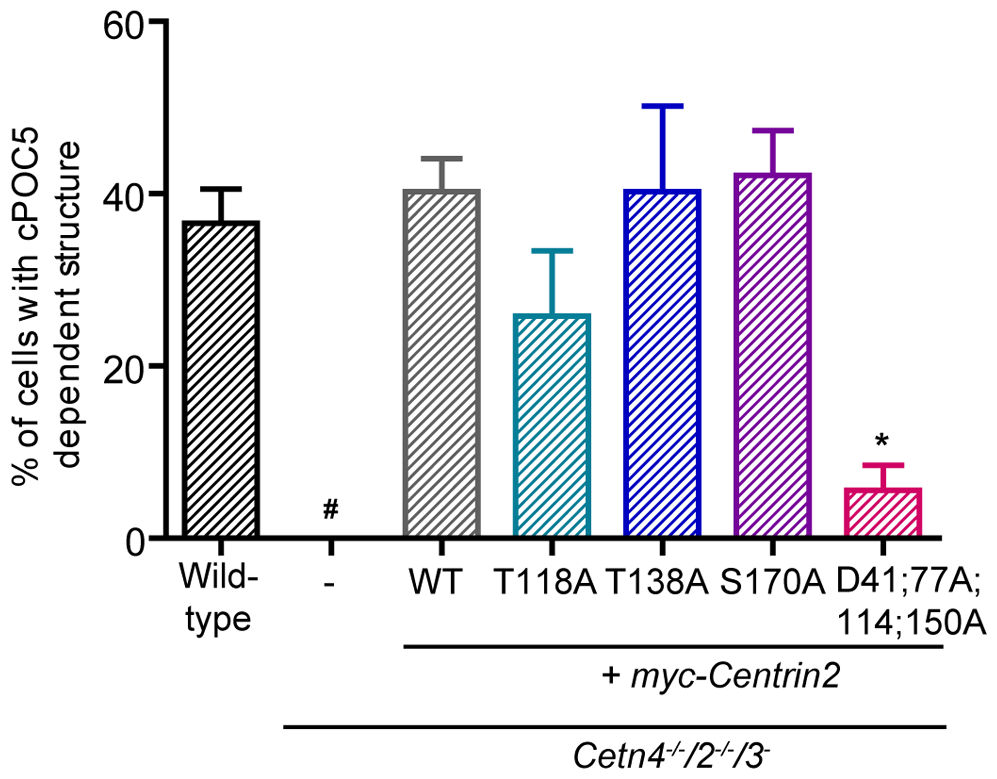
Requirement of centrin2 for cPOC5-induced structures. Histogram shows the mean % + s.d. of cPOC5-GFP-transfected cells of the indicated genotypes that formed a linear structure 24h after transfection. Datapoints were obtained from 3 separate experiments in which at least 150 transfectants were analysed. *, P<0.05 compared to wild-type cells by Kruskal-Wallis test and Dunn’s multiple comparison test. #, we observed no structures in the centrin null cells, so that a significance could not be calculated.

We next explored the dependency of this POC5-induced structure on centrin2 functioning by expressing cPOC5-GFP in the centrin2 mutant background. As shown in Figure 7, these structures were never observed in centrin-deficient cells. Expression of myc-centrin2 was sufficient to support the assembly of these structures, as was expression of the T138A and S170A mutants. However, the capacity to form the linear structures was greatly reduced in cells that expressed the T118A mutant and almost entirely lost in the calcium-binding mutant (Figure 7). Those structures that did form in the cells with only the calcium-binding mutant centrin2 were considerably reduced in size (data not shown). Together, our results suggest that centrosomal localisation of centrin2 is regulated by calcium binding and that, even though this capacity is not required for NER, it appears to be critical for centrin assembly into larger complexes.

## Discussion

Here, we have used site-directed mutagenesis of chicken centrin2 and its expression in centrin-deficient cells to probe the requirement for centrosome localisation in centrin activities. While we saw no effect of either T138A or S170A mutations on the centrosomal recruitment of myc-centrin2, we found that centrin2 localisation at centrosomes is dependent on the availability of a phosphorylatable T118 and functional calcium-binding EF-hands. Consistent with these observations, it was recently shown, by a similar site-directed mutagenesis approach, that recruitment of the centrin2 orthologue in *Tetrahymena thermophila*, TtCentrin1, to basal bodies depends on functional EF-hand motifs [43]. These findings suggest that centrin2 can localise to centrosomes without being phosphorylated at T138 or S170, but that the conserved MPS1 phosphorylation site at T118 and EF-hand function are necessary for efficient centrosome localisation. These conclusions expand the known roles of these regulatory elements of centrin2 [24,37,39,40].

Notably, despite the marked decline in centrosomal localisation we saw with the T118A and calcium-binding defective mutants of centrin2, both of these mutants showed the same capacity as wild-type centrin2 in rescuing the centrin null phenotype with respect to cell survival or centrosome amplification after UV treatment. Both the T138A or S170A mutants showed a similar capacity. These findings demonstrate that these mutations, while potentially perturbing centrin2 structure, are not so radical that they disrupt normal protein function. Furthermore, they show that the centrosomal localisation of centrin2 is dispensable for its role in NER. It thus seems likely that the nuclear localisation of centrin2 is an important determinant of NER efficiency. Several studies have indicated that this localisation depends on centrin2 interacting directly with XPC [36,56,57]. A role for centrin2 SUMOylation in regulating this interaction and thus, the movement of centrin2 into the nucleus for NER, was suggested from RNAi experiments that disrupted the SUMOylation machinery [58]. However, UV irradiation did not greatly alter the electrophoretic mobility of centrins [59], so that it is not clear to what extent the post-translational modification of centrin2, as opposed to its interactors, directs its function in NER.

In our analysis of the impact of centrin deficiency on XPC dynamics, we detected almost as rapid a recruitment of GFP-cXPC to DNA damage in centrin null cells as we observed in wild-type controls. This result is consistent with previous *in vitro* data that showed that centrin2 is dispensable for XPC binding to damaged DNA and the initiation of NER [33,34]. Nonetheless, a significant diminution of NER activity was seen in an *in vitro* reaction using a mutant form of XPC unable to interact with centrin2 [59]. Thus, together with these reports, our results suggest that centrin’s main activity in NER may be downstream of XPC recruitment to DNA lesions.

In a parallel set of experiments, we tested if the centrosomal recruitment of POC5 was dependent on centrins. Strikingly, almost half of the cPOC5-GFP transfectants assembled defined, linear structures that fully co-localised with centrin. In human U2OS cells, where we have access to more reagents, we also found that hPOC5 overexpression induces linear structures that contain centrin. While a small fraction of γ-tubulin and NEDD1 localised to the structures in approximately 50% of cells, we did not detect stabilised α-tubulin (acetylated/polyglutamylated tubulin) or any microtubule marker in them. PCM1 surrounds the structures, possibly reflecting the interaction of centrins with both POC5 and PCM1, rather than a functional consequence of the assembly [50,60]. The apparent lack of a microtubule basis to the POC5 structures was unexpected because their linear shape and because of the presence of components of the γ-TuRC, which functions in microtubule nucleation at the centrosome and which also plays a part in microtubule stability and dynamics [61]. By TEM imaging we were able to find some examples of over-elongated centriolar microtubules but the majority of the structures that we observed appeared to be large, dense protein aggregates.

The overexpression of CPAP (CenpJ) [51–53] or POC1 [55] and the depletion of CP110/Cep97 [54] have been also reported to induce the assembly of linear structures. The authors detected stabilized α-tubulin in these structures (post-translationally modified by acetylation and polyglutamylation), which is characteristic of centriolar and ciliary microtubules. Interestingly, in these studies, centrins were shown to completely co-localise with the linear structures in contrast to primary cilia, in which centrins are normally restricted to the basal bodies/centrioles. Conversely, the intraflagellar transport component IFT88 (Polaris) did not localise to the structures induced by CPAP overexpression or CP110 depletion, suggesting that these were not typical primary cilia [52]. Finally, TEM analysis revealed that the structures consisted of elongated centriolar microtubules with no transition zone or membranous surroundings. It was therefore concluded that these elongated structures were not genuine primary cilia but rather over-elongated centrioles [51,52].

While the identity of the POC5-induced linear structures is not fully established, it is noteworthy that those centrin2 mutants that failed to localise to centrosomes were compromised in their ability to form these structures but fully able to rescue the UV-hypersensitivity phenotype caused by centrin deficiency. This suggests that a distinct pool of centrin is involved in NER and that a multimeric assembly of centrins is not a requirement for NER. The assembly of centrin-POC5 structures does require the calcium-binding capacity of centrin2. The multiple molecules of the yeast centrin orthologue, Cdc31p, that assemble a filament on the α-helical scaffold of Sfi1p, do so independently of Ca^2+^ binding [62,63]. However, such a filament has the potential for developing a calcium-responsive contractile structure not seen in yeast, such as the striated rootlet or the ciliary basal body [63,64]. Scaffolded, multimeric assemblies of centrin2 at the distal end of the centriole may allow such dynamic structures to form.

## Materials and Methods

### Cell culture, transfections and clonogenic survival assays

Unless otherwise stated, cell culture reagents and drugs were from Sigma. DT40 cells were a gift from Shunichi Takeda (Kyoto University, Japan) [65] and were cultured as described [25]. Cells were transiently transfected with 5-10µg of endotoxin-free plasmid DNA using nucleofection (Kit T; programme B-23; Amaxa). Stable transfections were performed as previously described [66] using a GenePulser (BioRad) set to 550V/ 25µF with 15 µg of linearized plasmid DNA. 2mg/milliliter geneticin (Invitrogen) was used for antibiotic selection. Human U2OS and hTERT-RPE1 cells (ATCC) were grown in DMEM and DMEM-F12 respectively, both supplemented with 10% FBS and 1% P/S and incubated at 37^°^C with 5% CO_2_. U2OS and hTERT-RPE1 transient transfections were carried out using Lipofectamine 2000 (Invitrogen) according to the manufacturer’s instructions.

UV clonogenic survival assays were performed as described in [66], using Dulbecco’s Modified Eagle Medium F-12 with L-glutamine (DMEM/F-12, Gibco, Invitrogen), supplemented with 1.5% methylcellulose, 15% FBS, 1.5% chicken serum, 50µM β-mercaptoethanol and antibiotics. Briefly, 2.5x10^6^ cells were resuspended in 0.5ml of PBS and UV-irradiated with a 254 nm UV–C lamp at 23 J/m^2^/min (NU-6 lamp; Benda). Medium was then added and cells plated at different densities into the methylcellulose media. Colonies were scored 8-12 days after plating.

### Molecular biology

RNA was extracted from DT40 and hTERT-RPE1 cells using TRI reagent and reverse transcription was performed using SuperScript First-Strand (both Invitrogen). PCRs were performed using KOD Hot Start (Novagen/ Merck) with the following primers: *cXPC* (5’ region): 5’ GCTAGCATGGCGAGGAAGCGCAAAG 3’ and 5’ TCCCATCGTTGTCAAATCCCA 3’, *cXPC* (3’ region): 5’ GGACACACAATCGCTGGAGAC 3’ and 5’ TCTAGATCACAGTTTTTCAAAAGGAAACAAC 3’; *cPOC5*: 5’ ATAGCTAGCATGGAGCAACTTTGCCCTGTTTC 3’ and 5’ CGCGAATTCGGTCAACAACTTTTATTGACTG 3’; *hPOC5* (isoform 1) 5’ CGAGCTAGCATGTCATCAGATGAGGAGAAATAC 3’; *hPOC5* (isoform 2) 5’ CGAGCTAGCATGAAGTGGGAAGAATATGAAG 3’, both with the same reverse primer, 5’ CGCGAATTCGGTCAACCACTTTTATGGAATG 3’.

pGEM-T Easy (Promega) was used to clone cDNAs, which were sequenced before subcloning into pEGFP-C1 (XPC) or pEGFP-N1 (cPOC5/hPOC5). Site-directed mutagenesis was performed on the myc-centrin2 plasmid template [25] using KOD and complementary forward and reverse primers, prior to DpnI digestions and bacterial transformation. All plasmids were sequenced prior to use in transfection experiments.

### Immunofluorescence microscopy

DT40 cells were left to attach to poly-L-lysine-coated slides for 15 min while U2OS cells were grown directly on glass coverslips. Cells were then fixed in methanol with 5 mM EGTA (pre-chilled to -20^°^C) for 10 min. Alternatively, cells were plunged into 4% paraformaldehye in PBS for 10 min, then permeabilized in 0.15% Triton X-100 in PBS for 2 min. For CPD staining, DNA was denatured after fixation, but prior to permeabilization, by incubation in 0.07 M NaOH/PBS, for 8 min at RT. Thereafter, cells were stained as described in [25]. The following primary antibodies and respective dilutions were used in this study: Centrin2 (poly6288, Biolegend) at 1:250 (for DT40 cells); Centrin-2 (N-17, Santa Cruz) at 1:500; Centrin3 (3E6, Abnova) at 1:1000; mouse anti-myc 9E10 at 1:500 (for DT40 cells); Ninein (ab4447, Abcam) at 1:200; PCM-1 (817, a gift from Andreas Merdes [60]) at 1:10000; Pericentrin (ab4448, Abcam) at 1:500; α-tubulin (B512, Sigma) at 1:1000; γ-tubulin (GTU88, Sigma) at 1:500 (or 1:150 for DT40 cells); γ-tubulin (C-20, Santa Cruz) at 1:250; glutamylated tubulin (GT335, a gift from Carsten Janke [67]) at 1:300; acetylated tubulin (T6793, Sigma) at 1:1000; NEDD1 (a gift from A. Merdes [68]) at 1:300; Centrobin (ab70448, Abcam) at 1:500; ODF2/Cenexin (ab43840, Abcam) at 1:200; IFT88 (13967-1-AP, ProteinTech) at 1:200 (or 1:100 for DT40 cells); Rootletin (sc-67824, Santa Cruz) at 1:100; CPDs (CAC-NM-DND-001, Hölzel Diagnostika) at 1:1000. Fluorescently labeled secondary antibodies used were from Jackson Laboratories.

Cells were imaged on a DeltaVision integrated microscope system mounted on an IX71 microscope (Olympus) with a PlanApo N100x oil objective, (N.A. 1.40) and controlled by SoftWorx software (Applied Precision). Images were taken using a CoolSnap HQ2 camera (Photometrics), deconvolved using the ratio method, and saved as maximal intensity projections using SoftWorx. Cells labeled with CPD-specific antibodies were visualized by confocal microscopy using a Zeiss LSM510 Meta equipped with a Plan-Apochromat 63x oil objective lens (N.A. 1.40). Z-stacks were deconvolved and displayed as maximum intensity projections. High content imaging and analysis were performed using an Operetta high throughput imaging system (PerkinElmer) with 40x long working distance air objective (N.A. 0.6) and the Harmony software. Alternatively, cell counting was performed using an Olympus BX51 microscope, 100x objective (N.A. 1.35) with the Openlab software (Improvision).

Statistical analyses were performed with Prism v 5.0 (GraphPad).

### Live-cell visualisation of XPC recruitment

Cell lines that stably expressed GFP-cXPC were incubated for 3-12 hours in poly-D-lysine-coated dishes (MatTek) to adhere and the media was supplemented with 12.5mM HEPES (pH 7.5). Single cells were irradiated with a femtosecond ﬁber laser coupled to a confocal microscope that generates pulses of 775 nm (duration 250 fs, repetition rate 40 MHz), as described in [47]. The peak power density at the focal plane was 520 GW/cm^2^. Cells were irradiated along a single track with a length of 6 µm. Fluorescence measurements were carried out through a 40x oil immersion objective lens with a numerical aperture of 1.3 (EC-Plan-Neo-Fluar, Zeiss) using an Ar^+^ source (488 nm). The selected parameters, including laser power and magnification factor, were kept constant throughout all experiments. Images were taken every 5 seconds during a 3 min period. During the whole procedure, cells were kept on the microscope stage under controlled environmental conditions at 39.5 °C and 5% CO_2_. Recruitment kinetics were analyzed using a custom designed macro for ImageJ. To determine the relative GFP-cXPC enrichment at the damage sites, the mean intensity of the cXPC signal in the irradiated region was divided by the mean intensity of the whole nucleus. XPC accumulation within the irradiated area was detected automatically by selecting bright regions of pre-defined minimum size that localized inside a larger continuous elliptical shape corresponding to the trajectory of the laser. This quantification procedure was necessary to account for rotations of the nucleus after laser irradiation which could lead to discontinuities of the fluorescence signal along the irradiated line. The corresponding ImageJ macro is available at www.bioimaging-center.uni-konstanz.de.

### Immunoblotting

Whole-cell extracts were prepared using RIPA buffer (50mM Tris-HCl pH7.4, 1% NP-40, 0.25% sodium deoxycholate, 150mM NaCl, 1mM EDTA and protease inhibitor cocktail). Immunoblot analysis was performed using the following primary antibodies: mouse anti-myc 9E10 at 1:1500; γ-tubulin (C-20, Santa Cruz) at 1:1500; XPC (D-18, Santa Cruz) at 1:400; HRAD23B antibody (ab86781, Abcam) at 1:4000. Signals were acquired using a LAS-3000 Imager (Fujifilm) and quantified with MultiGauge v 2.2 (Fujifilm).

### Electron microscopy

After transfection with POC5-GFP constructs, human cells were prepared for transmission electron microscopy TEM as described in [69]. Samples were imaged with an H-7000 Electron Microscope (Hitachi) with an ORCA-HRL camera (Hamamatsu Photonics) and saved using AMT version 6 (AMT Imaging).

## Supporting Information

Figure S1 Immunofluorescence micrographs show examples of the structures induced by stable expression of hPOC5-GFP in U2OS cells. GFP is shown in the green channel, with relevant markers in red and DNA visualised with DAPI (blue). ‘Glut.’, Glutamylated. Blow-ups (2.5x) show the hPOC5-induced structures. Micrographs are representative of results obtained in at least 3 separate experiments. Scale bar, 5 µm.
(PDF)
