# Supplementary figures and images for: Calcium-Binding Capacity of Centrin2 Is Required for Linear POC5 Assembly but Not for Nucleotide Excision Repair
Source: PLoS One. 2013 Jul 2;8(7):e68487. doi: 10.1371/journal.pone.0068487 (PMC3699651; doi:10.1371/journal.pone.0068487)

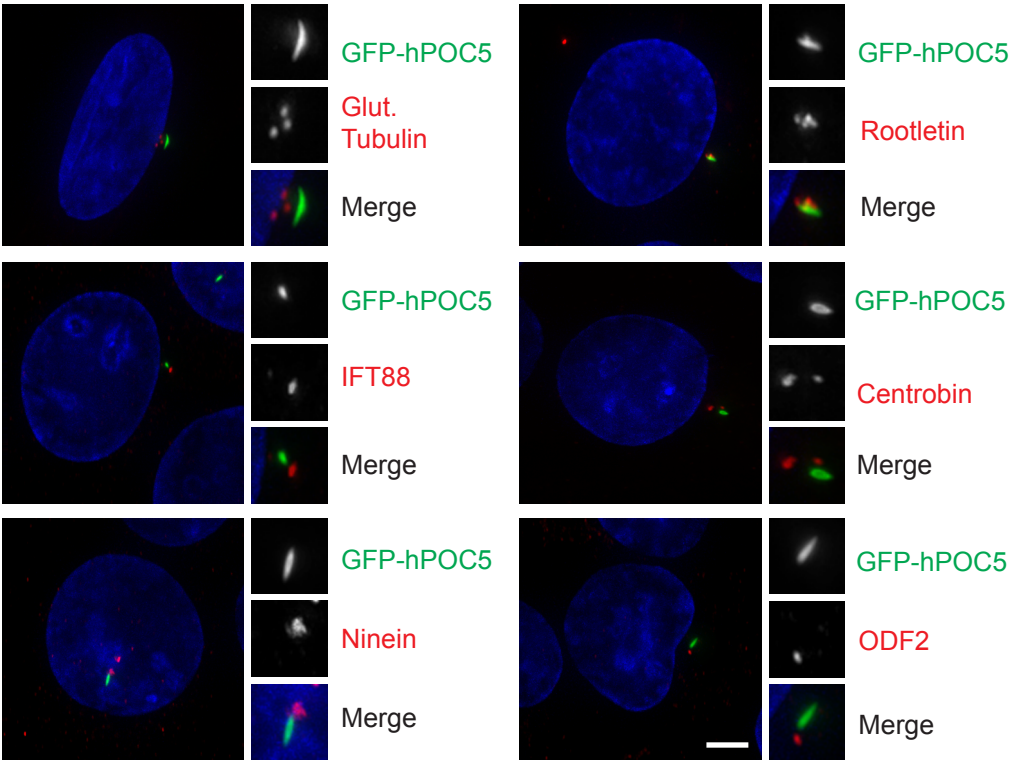

Supplement: Figure S1 — Immunofluorescence micrographs show examples of the structures induced by stable expression of hPOC5-GFP in U2OS cells. GFP is shown in the green channel, with relevant markers in red and DNA visualised with DAPI (blue). ‘Glut.’, Glutamylated. Blow-ups (2.5x) show the hPOC5-induced structures. Micrographs are representative of results obtained in at least 3 separate experiments. Scale bar, 5 µm. (PDF) [file pone.0068487.s001.pdf]
